# Mitogen-activated protein kinase kinase 5 (MKK5)-mediated signalling cascade regulates expression of iron superoxide dismutase gene in *Arabidopsis* under salinity stress

**DOI:** 10.1093/jxb/erv305

**Published:** 2015-07-01

**Authors:** Yu Xing, Wei-hua Chen, Wensuo Jia, Jianhua Zhang

**Affiliations:** 1 Beijing Key Laboratory for Agricultural Application and New Technique and College of Plant Science and Technology, Beijing University of Agriculture, Beijing, China; 2 Institute of Agro-Products Processing Science & Technology CAAS, Beijing, China; 3 College of Agronomy and Biotechnology, China Agricultural University, Beijing, China; 4 School of Life Sciences, State Key Laboratory of Agrobiotechnology, and Shenzhen Research Institute, The Chinese University of Hong Kong, Hong Kong, China

**Keywords:** *Arabidopsis*, FSD, iron superoxide dismutase, mitogen-activated protein kinase kinase 5, mitogen-activated protein kinase 6, salt stress

## Abstract

Superoxide dismutases (SODs) are involved in plant adaptive responses to biotic and abiotic stresses but the upstream signalling process that modulates their expression is not clear. Expression of two iron SODs, *FSD2* and *FSD3,* was significantly increased in *Arabidopsis* in response to NaCl treatment but blocked in transgenic *MKK5*-RNAi plant, *mkk5*. Using an assay system for transient expression in protoplasts, it was found that mitogen-activated protein kinase kinase 5 (MKK5) was also activated in response to salt stress. Overexpression of *MKK5* in wild-type plants enhanced their tolerance to salt treatments, while *mkk5* mutant exhibited hypersensitivity to salt stress in germination on salt-containing media. Moreover, another kinase, MPK6, was also involved in the MKK5-mediated iron superoxide dismutase (FSD) signalling pathway in salt stress. The kinase activity of MPK6 was totally turned off in *mkk5*, whereas the activity of MPK3 was only partially blocked. MKK5 interacted with the MEKK1 protein that was also involved in the salt-induced *FSD* signalling pathway. These data suggest that salt-induced *FSD2* and *FSD3* expressions are influenced by MEKK1 via MKK5MPK6-coupled signalling. This MAP kinase cascade (MEKK1, MKK5, and MPK6) mediates the salt-induced expression of iron superoxide dismutases.

## Teaser

Salt-induced expressions of iron superoxide dismutases (FSD2 and FSD3) are regulated by MKK5 that interacts with MEKK1 via the MKK5-MPK6-coupled signalling cascade.

## Introduction

Reactive oxygen species (ROS) have long been known to be harmful to many cellular processes, however, a transient increase in ROS can also act as a signal that mediates the regulation of various cellular activities. Examples include responses to biotic or abiotic stresses, cell death, stomatal movement or root hair development (Gill and Tuteja, 2010; Acharya *et al.*, 2013; Suzuki *et al.*, 2013). As a first line of defence against ROS, the enzyme superoxide dismutase (SOD) catalyses the initial step in the AsadaHalliwell pathway in chloroplasts and converts superoxide to hydrogen peroxide and molecular oxygen (Bowler *et al.*, 1994; Allen, 1995). SOD enzymes are classified into three groups according to their metal co-factor: iron SOD (FeSOD), manganese SOD (MnSOD), and copper-zinc SOD (Cu-Zn SOD) and are located in different cellular compartments (McKersie *et al.*, 2000).

There is growing evidence to suggest that SODs and other anti-oxidative enzymes are closely associated with plant biotic or abiotic stress tolerance, although the signalling processes involved in stress-induced gene expression of SODs are largely unknown (Miller *et al.*, 2008, van Breuseqem *et al.*, 2008; Li *et al.*, 2013; Xing *et al.*, 2013). Overexpressing some SODs has been demonstrated to enhance diverse stress tolerances, such as to low temperature and high light (McKersie *et al.*, 2000; Huang *et al.*, 2012) and other abiotic stresses (Gill and Tuteja, 2010). A decrease in MnSOD leads to reduced root growth and affects tricarboxylic acid cycle flux and mitochondrial redox homeostasis (Morgan *et al.*, 2008). *SaFeSOD* cloned from *Sonneratia alba*, a highly salt tolerant mangrove tree, is expressed in leaf, stem, flower, fruit, and root tissues with the highest expression in leaf tissues (Wang *et al.*, 2013). Under nitrogen supplemented conditions, overexpression of either SOD protein, especially FeSOD, conferred significant tolerance against oxidative stress (Raghavan *et al.*, 2011). Nevertheless, the signalling processes of stress-induced gene expression of SODs are still largely unknown (McKersie *et al.*, 2000; Miller *et al.*, 2008; van Breusegem *et al.*, 2008).

Several lines of evidence suggest that the MAPK signalling cascade is, at several levels, involved in plant biotic or abiotic stress-induced signalling and indeed, most plant MAPKs investigated to date have been linked to stress responses (Xing *et al.*, 2008, 2013; Ding *et al.*, 2013; Li *et al.*, 2013; Sheikh *et al.*, 2013). In studies with alfalfa (*Medicago sativa*), stress-induced MAPKK (SIMKK) was isolated in a yeast two-hybrid interaction screen using the MAP kinase SIMK as a bait and the specific activation of SIMK by SIMKK was subsequently observed upon salt stress in protoplasts, supporting the function of SIMKK as an upstream activator of SIMK in this pathway (Kiegerl *et al.*, 2000). A complete *Arabidopsis* MAPK signalling cascade pathway (MEKK1, MKK4/MKK5, and MPK3/6) has been identified and shown to be involved in the induction of expression of the WRKY22/WRKY29 transcription factors by the bacterial protein flagellin (Asai *et al.*, 2002). Moreover, *Arabidopsis* MKK2 has been shown to be activated by MEKK1 and to increase freezing and salt tolerance by activating its direct targets MPK4 and MPK6, as well as the expression of other stress-induced marker genes (Teige *et al.*, 2004). Previous reports have also shown that hydrogen peroxide-mediated stress signalling requires MAP kinase cascades (Kovtun *et al.*, 2000; Kumar and Klessig, 2000; Samuel *et al.*, 2000; Zhang *et al.*, 2006; Xing *et al.*, 2008), suggesting an important role for MAPK signalling in the generation of ROS and detoxification.

The involvement of *Arabidopsis* MKK5 in the MAP kinase cascade was suggested by its response to flagellin-induced signalling (Asai *et al.*, 2002). Furthermore, *Arabidopsis* MKK5 has been shown to be involved in hydrogen peroxide-mediated cell death and oxidative stress (Ren *et al.*, 2002; Xing *et al.*, 2013). Taken together, these findings suggest that *Arabidopsis* MKK5 might be involved in both abiotic and biotic stress signalling. However, the exact roles and detailed mechanisms of MKK5-mediated signalling remain unknown.

To elucidate the potential role of MAPK signalling cascades in salt stress responses, an *Arabidopsis* protoplast transient expression system was used, in which transcription of *FeSOD* genes is induced by NaCl, allowing the roles of MAPK cascade components to be systematically evaluated. Using this system, MEKK1 was identified via the MKK5MPK6-coupled signalling associated with salt-induced FeSOD expression. These data suggest that this signalling pathway functions in response to salt stress and could potentially be utilized to enhance salt tolerance in crops.

## Materials and methods

### Plant materials and stress treatments

*Arabidopsis* thaliana (L.) plants were kept in a growth chamber at 222 C with 16h light and 8h dark and a relative humidity of 90%. RNAi gene-silenced plants of MKK5 and plants from MKK5-RNAi lines, MKK57, named *mkk5* were generated and chosen for this study (Xing *et al.*, 2013). For NaCl treatment of germination, wild-type and mutant seedlings growing on MS agar plates (MS complete medium with 30g/l sucrose and 7g/l agar) were transferred onto filter paper saturated with 150mM NaCl and the plants were incubated under light for various times. For NaCl treatment of protoplasts, isolated protoplasts from *Arabidopsis* leaves were subjected to a final NaCl concentration of 150mM for 10min.

### Plasmid construction

#### -glucuronidase-tagged reporter vector construction

For construction of the vector containing -glucuronidase (GUS), GUS was substituted for GFP in the pHBT95-GFP vector by using the *Nco*I and *Not*I restriction sites. This vector was named pHBT95-GUS. The promoter regions of the iron superoxide dismutase (FSD)1, FSD2, and FSD3 genes were amplified by PCR from *Arabidopsis* (Col-0) genomic DNA (FSD1: forward primer, 5-ATATGGTTT ACCCATCTTAATTT-3, reverse primer, 5-TCTTTGTAATTG AAGCTGCACATT-3; FSD2: forward primer, 5-TAAAATTAA AACATTAAATTATAT-3, reverse primer, 5-CTTCACTCAAAG CGTTACTGATTAT-3, and FSD3: 5-AGTTCCTCCCACTGT TGTCGTCA-3, 5-TAGGTAAGATGATTAAATCGACAG-3) and were substituted for the cauliflower mosaic virus (CaMV) 35S promoter using the *Xho*I and *Apa*I sites, to yield pFSD1-GUS, pFSD2-GUS, and pFSD3-GUS. The pMPK3-GUS, pMPK4-GUS, pMPK6-GUS, and pMKK5-GUS vectors were constructed using the same method.

#### Effector vector construction

*Arabidopsis* MAPKK cDNAs were: MKK2 (At4g29810), MKK4 (At1g51660), and MKK5 (At3g21220). The MAPKs were MPK2 (At1g59580), MPK3 (At3G45640), MPK4 (At4g01370), MPK6 (At2g43790), MPK7 (At2g18170), and MPK9 (At3g18040). All cDNAs were amplified by PCR from *Arabidopsis* (Col-0 or mutants), cloned into the pENTR-TOPO cloning vector (Invitrogen, Carlsbad, CA, USA) and verified by sequencing. PCR products were inserted into the pGWB5-DHA vector containing a double HA epitope tag, the 35S promoter, and the NOS terminator.

### Total RNA extraction, semi-quantitative reverse transcription-PCR and northern blot analysis

Seedlings (mutant and wild-type plants) were grown for 2 weeks on MS agar plates under continuous light and then treated with NaCl as described above. Total RNA was extracted using the RNeasy Plant Mini Kit (Qiagen, Hilden, Germany) according to the manufacturers instructions and stored at 80 C until further use. Total RNA was used as a template for first-strand cDNA synthesis using the SuperScript II First-Strand Synthesis System for reverse transcription (RT)-PCR (Invitrogen). The PCR volume was 25 l, containing 100ng of each primer, 2mM each dNTP, 0.5 l cDNA, and 0.75 units of *Taq* DNA polymerase (Invitrogen), and the reactions were run in a PTC-100 Programmable Thermal Controller (MJ Research, Watertown, MA, USA). PCR products were separated on 1.5% agarose gels.

For northern blot analysis, 15 g of total RNA was fractionated on a 1.0% formaldehyde-containing agarose gel with an RNA molecular weight marker (Promega Corp., Madison, WI, USA) and blotted onto a nylon membrane (Hybond-N, Amersham Pharmacia Biotech, Aylesbury, UK) overnight at room temperature. Equal loading of RNA was confirmed using ethidium bromide staining of the gel prior to transfer. Probes were labelled with [-^32^P] dCTP using a random-primed DNA labelling kit (Megaprime, Amersham) and hybridization was performed according to the manufacturers recommendations.

### Protoplasts preparation and transformation

*Arabidopsis* protoplasts were isolated using a modified protocol from Abel and Theologis (1994). Leaves from 4-week old *Arabidopsis* plants were washed in distilled water and incubated with an enzyme solution containing 1% Cellulase R10, 0.25% Macerozyme R10 (both from Yakult, Tokyo, Japan), and 0.4M mannitol for about 34h at 23 C with gentle shaking (50rpm). The protoplast suspension was filtered through a net with a pore diameter of 150 M. After centrifugation (60 *g*, 2min), the supernatant was discarded and the cells were re-suspended in washing solution and washed twice in washing buffer containing Magma solution. The concentration of protoplasts was determined and the viability of the cells was verified by staining with FDA (fluoresce in diacetate) and subsequently by fluorescent microscopy (Zeiss Axioskop) (Nunberg and Thomas, 1993). Plasmid DNA solutions were adjusted with 0.5M mannitol and mixed with 510^5^ protoplasts. After addition of an equal volume of PEG 6000 solution, the suspension was incubated at room temperature for 10min and washed with washing and incubation (WI) solution before being re-suspended in WI solution. The transformed cells were incubated in the dark (23 C, 30rpm, overnight) and the suspensions were divided into different tubes, treated with NaCl solution and incubated for the appropriate times.

### Assay for transient GUS activity

The suspensions of protoplasts were centrifuged (60 *g*, 2min) and washed with 0.5M mannitol. Pellets were dissolved in 105 l extraction buffer and extracts were vortexed and kept at 80 C for at least 1h or in liquid nitrogen for several minutes. GUS activity was quantified according to Ftterer *et al.* (1990) and a modified protocol for suppressing endogenous GUS activity (Kosugi *et al.*, 1990). The protein concentration was measured according to the manufacturers instructions (Bio-Rad, Hercules, CA, USA) with BSA as a standard. GUS activity was measured in the supernatant after adding the substrate 4-methyl-umbelliferyl--d-glucuronide (MUG). MUG was hydrolysed by GUS protein into 4-methyl-umbelliferone or 7-hydroxy-4-methyl-cumarin (4-MU) and glucuronide, and GUS activity determined in a dark micro-titre plate (Nunc GmbH & Co. KG, Wiesbaden, Germany) using the HTS 7000 plus Bioassay Reader (emission, 460nm; excitation, 365nm). GUS activity was normalized according to the expression derived from pHBT95-GUS with no fusion protein (average 204 pmol of 4-MU produced per minute per microgram of protein obtained from six independent experiments).

### Expression and purification of GST fusion proteins

Full-length *Arabidopsis* MKK5, MPK3, MPK4, and MPK6 cDNAs were obtained using RT-PCR, cloned into the pENTR-TOPO cloning vector (Invitrogen) and sequenced. The *Escherichia coli* Strain BL-21 codon plus (Stratagene, LA Jolla, CA, USA) was transformed with the expression constructs, which was prepared by subcloning the genes into pGEX-6P-1 vector (Amersham Pharmacia Biotech). Growth of bacteria and isolation of recombinant GST fusion protein was as described in Matsuoka *et al.* (2002).

### Protein extraction, immunoprecipitation and kinase activity assay

The following steps were carried out at 4 C unless otherwise stated. Plant tissues (3:1 buffer volume:fresh weight) were homogenized with a pestle and mortar in 100mM Tris-HCl buffer (pH 8.0) containing 2mM EDTA, 5mM DTT, 10% glycerol, 1mM phenylmethylsulphonyl fluoride (PMSF), and 0.3 M aprotinin. The homogenate was filtered through four layers of muslin cloth and centrifuged at 12,000 *g* for 40min. The supernatant was desalted with a Sephadex G-25 column equilibrated with buffer suitable for the individual enzymes. The desalted supernatants were stored in aliquots at 80 C. The protein concentration was determined using the protein assay kit (Bio-Rad) with BSA as a standard.

Protein extracts (0.5mg) were incubated with 50 l antibody at 4 C overnight. Protein G-agarose beads (50 l) were added and incubated for 2h at 4 C. The proteinantibody complex on the beads was collected and washed three times in ice-cold PBS and before re-suspension in protein sample buffer.

The coding regions of MPK3, MPK4, and MPK6 were cloned into the pGEX-6P-1 vector and expressed as GST fusion proteins in BL21 codon plus *E. coli* cells (see above). Kinase inactive GST-MPK fusion proteins were generated by exchanging a conserved lysine reside in the ATP binding domains to methionine and arginine using the Quick Change kit (Stratagene). The point mutations were performed as described by Teige *et al.* (2004). Inactive GST-MPK (2 g) was incubated in 20 l of kinase reaction buffer (50mM Tris, pH 7.5, 10mM MgCl_2_, 1mM DTT, 0.1mM ATP, and 8 Ci of ^32^P-ATP) with immunoprecipitated GST fused to MKK5 from protoplasts. Kinase reactions were stopped after 30min by adding 4 l SDS loading buffer and heating for 5min at 95 C. Reaction products were analysed by SDS-PAGE, autoradiography, and Coomassie brilliant blue R250 staining.

In-gel kinase assays were performed essentially as described by Katou *et al.* (1999) with some modifications. Briefly, samples (20 g) of total protein or immunoprecipitate from 400 g of total protein were separated on a 10% SDS-polyacrylamide gel polymerized in the presence of 0.25mg/ml bovine brain myelin basic protein (Sigma). After electrophoresis, SDS was removed by washing the gel in buffer (25mM Tris-HCl pH 8.0, 0.1mM Na_3_VO_4_, 5mM NaF, 0.5mM DTT, 0.5mg/ml BSA, and 0.1% Triton X-100) three times (30min each) at room temperature. After 1h denaturation in a denaturing buffer containing guanidine, the kinases were allowed to renature overnight at 4 C with five changes of renaturing buffer (25mM Tris, pH 7.5, 1mM DTT, 0.1mM Na_3_VO_4_, and 5mM NaF). The phosphorylation of myelin basic protein was performed in 30ml reaction buffer (25mM Tris, pH 7.5, 0.1mM Na_3_VO_4_, 12mM MgCl_2_, 2mM EGTA, and 1mM DTT) with a pre-reaction for 30min, then 0.2 M ATP and 50 Ci of ^32^P-ATP in reaction buffer was added and the reactions incubated at room temperature for 6090min. The gel was then transferred into washing buffer at room temperature for at least 6h with six changes of buffer. Finally, the gel was dried on filter paper and autoradiographed.

### Immunocomplex kinase assays

The method for kinase assays has been described by Xing *et al.* (2008, 2013). Kinase inactive MPK6-GST protein (1 g) immunoprecipitated from *Arabidopsis* protoplast using anti-GST was incubated with MKK5 immunoprecipitated from *Arabidopsis* seedlings using anti-MKK5 in the kinase reaction mixture [20 l of kinase reaction buffer containing 50mM Tris (pH 7.5), 1mM DTT, 10mM MgCl_2_, 0.1mM ATP, and 9.710^3^Bq of ^32^P-ATP] for 30min at room temperature. The reaction was stopped after 30min by adding 4 l of SDS loading buffer and heating for 5min at 95 C. Reaction products were analysed by autoradiography after SDS-PAGE. MPK3-GST, MPK4-GST, and MPK6-GST fusion proteins were generated by exchanging a conserved lysine residue in the ATP binding domains to methionine and arginine using the Quick Change kit (Stratagene). The point mutations for MPK6 were K92M and K93R (Teige *et al.*, 2004).

### Generation of MKK5 overexpressing plants

Full-length *Arabidopsis* MKK5 cDNA was obtained by RT-PCR, cloned into the pENTR-TOPO cloning vector (Invitrogen) and sequenced. After the LR reaction, MKK5 cDNA was inserted into the pGWB5-DHA vector, and the vector named pGWB5-DHA-MKK5. Transgenic *Arabidopsis* plants expressing the DHA-tagged MKK5 under control of the CaMV 35S promoter were generated using the floral dip method (Clough and Bent, 1998) and either Col-0 wild-type plants or *mkk5* plants. Transformed plants were selected for growth on kanamycin containing media. Second generation plants were used for experiments.

### Determination of O_2_ production and SOD activity in *Arabidopsis* leaves

O_2_ production in *Arabidopsis* leaves of treated and control plants was determined according to the method of Able *et al.* (1998) by monitoring the reduction of 3-[1-(phenylamino-carbonyl)-3,4-tetrazolium]-bis(4-methoxy-6-nitro) benzenesulphonic acid hydrate (XTT) in the presence of O_2_. Leaves (1g) were frozen in N_2_, ground, and then homogenized with 5ml of 50mM TRIS-HCl buffer (pH 7.5) and centrifuged at 5000 *g* for 10min. The reaction mixture of 1ml contained 50 g of supernatant proteins, 50mM TRIS-HCl buffer (pH 7.5), and 0.5mM XTT. The reduction of XTT was determined at 470nm for 5min. Corrections were made for the background absorbance in the presence of 50 units SOD. O_2_ production rate was calculated using an extinction coefficient of 2.1610^4^ M^1^ cm^1^. SOD (EC 1.12.1.11) was measured in the leaves as previously described (Xing *et al.* 2013).

### Yeast two-hybrid interactions

Yeast two-hybrid interactions were performed using the ProQuest^TM^ Two-Hybrid System (Invitrogen) according to the manufacturers instructions.

## Results

### Salt-Induced FeSODs in *Arabidopsis*

**Fig. 1.**
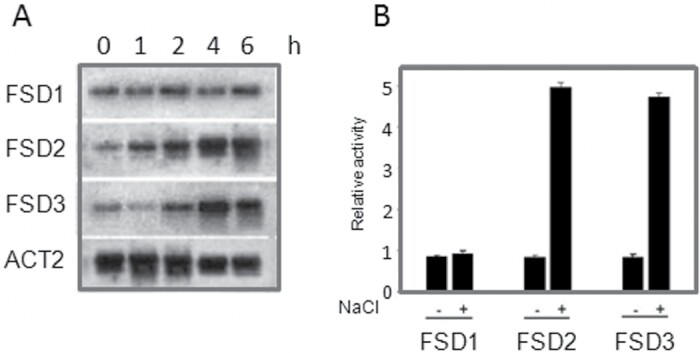
*FSD1*, *FSD2*, and *FSD3* expression following NaCl treatment. (A) Northern blot analysis of *FSD1*, *FSD2*, and *FSD3* expression. *Arabidopsis* tissues from different points of the time course were used for RNA gel blot analysis. Total RNA was extracted from wild-type plants without stress treatment (control) or subjected to 150mM NaCl, and incubated for different times. The actin gene *ACT2* was used as a loading control. (B) Relative *GUS* activity, driven by the *FSD1*, *FSD2*, or *FSD3* promoters, showed the response to NaCl. The *FSD1*, *FSD2*, and *FSD3* promoters were fused to *GUS* and tested for their response to NaCl in a protoplast transient expression assay. All experiments were repeated at least three times with similar results.

Salt-induced expression of FSD genes in *Arabidopsis* was investigated by northern blot analysis using RNA extracted from leaves. Treatment of plants with 150mM NaCl led to a significant increase in the expression of *FSD2* and *FSD3*, with *FSD2* responding earlier than *FSD3*. The *FSD2* gene transcript increased within 1h of treatment, peaked at 4h, and remained high until 6h, while *FSD3* transcription was induced within 2h and remained high until 6h. In contrast, *FSD1* transcript level was not altered by NaCl treatment (Fig.1A).

To further characterize *FeSOD* gene activation, the promoters of *FSD1*, *FSD2*, and *FSD3* (1.2kb before the ATG) were fused to the -glucuronidase reporter gene (*GUS*) and tested for response to NaCl in transiently transfected protoplasts. Consistent with the northern blot data obtained with the endogenous genes, the *FSD2* and *FSD3* promoters but not those of *FSD1* were activated by NaCl (Fig. 1B). Although the *FSD1* gene was expressed, it was not induced by NaCl. An earlier report indicated that *FSD1* transcription is under the control of a circadian clock (Kliebenstein *et al.*, 1998); however, these results did not show that *FSD2* or *FSD3* are controlled by a circadian clock mechanism (data not shown).

### Salt-induced FSD signalling operates through MKK5

**Fig. 2.**
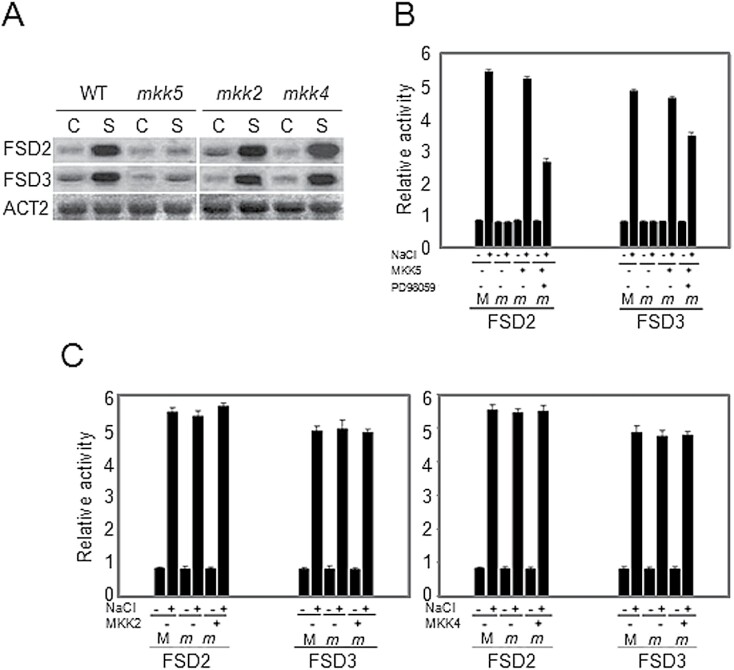
Involvement of *MKK5* in salt stress responses. RNA gel blot analysis of *FSD2* and *FSD3* transcript levels in response to salt stress in wild-type *Arabidopsis* plants, or the *mkk2*, *mkk4*, and *mkk5* mutants. Total RNA was extracted from wild-type (WT) or mutant plants without stress treatment (control, C) or subjected to NaCl (150mM) for 4h (S). The actin gene *ACT2* was used as a loading control. (B) Relative *GUS* activity driven by *FSD2* or *FSD3* promoter, respectively, showed the response of *FSD2* and *FSD3* to NaCl. The promoters of the *FSD2* and *FSD3* genes were fused to the *GUS* reporter gene and tested for their response to NaCl in transiently transformed protoplasts from wild-type plants or the *mkk5* mutants. The *MKK5* genes were cloned and co-transformed into protoplasts of the *mkk5* mutant. Transformed protoplasts were pre-incubated 1h with 5 M PD98059 before treatment with 150mM NaCl. Protoplasts were isolated from wild-type (M) and the *mkk5* mutant (*m*) leaves. (C) Relative *GUS* activity driven by *FSD2* or *FSD3* promoter, respectively, showed the response of *FSD2* and *FSD3* to NaCl. The promoters of the *FSD2* and *FSD3* genes were fused to the *GUS* reporter gene and tested for their response to NaCl in transiently transformed protoplasts from wild-type plants or the *mkk2* and *mkk4* mutants. The *MKK2* and *MKK4* genes were cloned and co-transformed into protoplasts of the *mkk2* or *mkk4* mutant, respectively. Protoplasts were isolated from wild-type (M) and mutant (*m*) leaves. All experiments were repeated at least three times with similar results.

In a previous study all the 10 known mutants of the *MAPKK* family in *Arabidopsis* were screened for downstream signalling components (Xing *et al.*, 2007, 2008, 2013). These *MAPKK* mutants were tested for *FSD* gene expression under salt stress in this study. Interestingly, the salt-activated gene expression of *FSD2* and *FSD3* that is apparent in wild-type plants was absent in the *MKK5*-RNAi plants (*mkk5*) but still present in *MKK4*-RNAi lines (*mkk4*). MKK4 is classified in the same subfamily as MKK5 and has the highest degree of DNA sequence homology to MKK5. Although MKK2 has also been shown to respond to NaCl-induced salt stress (Teige *et al.*, 2004), the NaCl-induced increases of *FSD2* and *FSD3* transcripts were not blocked in *MKK2* mutant, *mkk2* plants (Fig. 2A). These results suggest that MKK5 is a strong candidate for mediating the salt-induced *FSD* expression.

To determine whether the *FSD2* and *FSD3* promoters were activated by salt through MKK5 signalling, protoplasts isolated from *mkk5* leaves were transiently transfected with a pFSD2-*GUS* and a pFSD3-*GUS* reporter construct. In the *mkk5* protoplasts, neither of the two promoters was activated by NaCl (Fig. 2B), although transient expression of wild-type *MKK5* restored NaCl-induced activation of the promoters in the *mkk5* mutant protoplasts (Fig. 2B). Consistent with these results, PD98059, a broad-spectrum MAPK kinase inhibitor, partially impaired the ability of NaCl to activate the *FSD2* and *FSD3* promotersmost notably that of *FSD2* (Fig. 2B).

It was observed that the *FSD2* and *FSD3* promoters were activated by salt in a protoplast assay using protoplasts isolated from leaves of the *mkk2* and *mkk4* RNAi lines. Co-transformation of *MKK2* or *MKK4* with *FSD2* or *FSD3* promoters into protoplasts from *mkk2* or *mkk4* mutant leaves, respectively, showed no difference in activation patterns compared with wild-type (Fig. 2C). Together these results suggest that neither salt-activated MKK2 nor MKK4, which is homologous to MKK5, is involved in salt-induced FSD signalling.

### The involvement of specific MAPKs in NaCl-induced FSD signalling

**Fig. 3.**
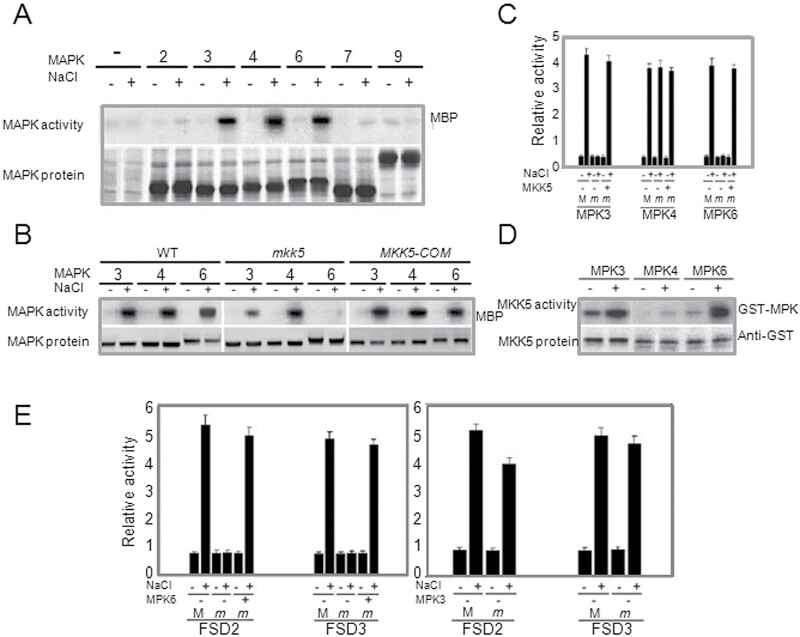
NaCl activates *MPK3* and *MPK6* through *MKK5*. (A) *MAPK* activation with or without NaCl 10min and expression of each MAPK. (B) Salt-triggered activation of *MPK3*, *MPK4*, and *MPK6* in *Arabidopsis*. Kinetics of *MPK3*, *MPK4*, and *MPK6* activation were measured in wild-type and *mkk5* mutant plants in response to salt stress. *MPK3*, *MPK4*, and *MPK6* were immunoprecipitated from leaf cell extracts of salt-induced plants. MPK activity was measured in immunocomplex kinase assays using myelin basic protein (MBP) as a substrate and the levels of MPK3, MPK4, and MPK6 proteins were measured by western blot analysis. (C) Relative *GUS* activity driven by the *MPK3*, *MPK4*, or *MPK6* promoter, respectively, showed *MPK3*, *MPK4*, and *MPK6* response to NaCl in transiently transformed protoplasts of wild-type (M) and *mkk5* mutant (*m*). The *MKK5* genes were cloned and co-transformed into protoplasts of the *mkk5* mutant. (D) *In vitro* phosphorylation of MPK3, MPK4, and MPK6 by active MKK5. GST-tagged MKK5 was immunoprecipitated from *Arabidopsis* protoplasts before and 10min after salt stress treatment. Immunoprecipitated MKK5 was subsequently used for phosphorylation of recombinant kinase inactive GST-MPK3, GST-MPK4, and GST-MPK6, respectively. Phosphorylation of MPKs was analysed by autoradiography after SDS-PAGE. MKK5 protein was detected using a GST antibody. (E) Relative *GUS* activity driven by the *FSD2* and *FSD3* promoters showed the *FSD2* and *FSD3* response to NaCl in transient expression assays using protoplasts from wild-type (M) or *mpk3* and *mpk6* mutants (*m*). The MKK5 genes were cloned and co-transformed into protoplasts of the *mkk5* mutant. All experiments were repeated at least three times with similar results.

To determine which MAPKs are involved in salt-induced FSD signalling, six MAPKs were targeted that collectively represent four of the five MAPK subfamilies, and which may exhibit distinct functions based on sequence homology analysis (Mizoguchi *et al.*, 2000, Tena *et al.*, 2000; Hamel *et al.*, 2006). The coding sequences of these *MAPKs* were each introduced into the pHBT95 vector in frame with the coding sequence of a GST, transiently expressed in protoplasts, immunoprecipitated with an anti-GST antibody, and tested for *in vitro* MAP kinase activity. MPK3, MPK4, and MPK6 all showed strong activation following NaCl treatment (Fig. 3A), while MPK7 showed a small activation but an overall weak expression. MPK2 and MPK9 did not show an altered expression (Fig. 3A).

To investigate the molecular mechanism of MKK5 action in salt-induced FSD signalling, MPK3, MPK4, and MPK6 activation was analysed in wild-type and *mkk5* plants. NaCl activation of MPK3 and MPK6, which belong to the same subfamily, but not MPK4, in wild-type, decreased in the *mkk5* mutant protoplasts unless functional MKK5 was co-expressed in the *mkk5* mutant (Fig. 3B). The activation of MPK6 was absent in *mkk5* mutant protoplasts, whereas the activation of MPK3 was decreased, suggesting that MPK3 is not regulated to the same extent by MKK5 in salt-induced FSD signalling. To investigate whether the *MPK3*, *MPK4*, and *MPK6* genes were activated transcriptionally in wild-type and the *mkk5* mutant, the promoters of *MPK3*, *MPK4*, and *MPK6* genes were fused to *GUS* and tested for their response to NaCl in transiently transfected protoplasts of wild-type and the *mkk5* mutant. Consistent with the MAPK activity results, NaCl activation of *MPK3* and *MPK6* promoters, but not the *MPK4* promoter, was blocked in *mkk5* and restored when co-expressed with *MKK5* (Fig. 3C). Together, these results suggest that MPK3 and MPK6, but not MPK4, are involved in MKK5-mediated salt-induced FSD signalling and that another signal is required to regulate NaCl-induced MPK4 signalling.

To investigate the phosphorylation targets of MKK5 *in vitro*, recombinant kinase inactive GST fusion proteins of MPK3, MPK4, and MPK6 were expressed and purified. MKK5 was expressed under the control of the 35S CaMV promoter, immunoprecipitated from transiently transformed protoplasts and tested for its ability to phosphorylate MPK3, MPK4, and MPK6 *in vitro* after activation by salt stress for 10min. Consistent with the above results, both MPK3 and MPK6, but not MPK4, were phosphorylated by MKK5 (Fig. 3D).

To directly test the role of MPK3 and MPK6 in salt-induced FSD signalling, protoplasts isolated from *mpk3* or *mpk6* mutant leaves were transiently transformed with the *FSD2*-*GUS* and *FSD3*-*GUS* reporter constructs. In the *mpk6* mutant protoplasts, neither of the two promoters was activated by NaCl and the relative *GUS* activity of the two promoters was restored when co-expressing *MPK6* in *mpk6* mutant protoplasts. In contrast, the two promoters maintained the same activated level in the *mpk3* mutant protoplasts as the wild-type (Fig. 3E). Thus, it appears that while the salt-induced expression of *FSD2* and *FSD3* requires MPK6, it does not require MPK3, even though both of them can be activated by MKK5.

### NaCl activation of MKK5

**Fig. 4.**
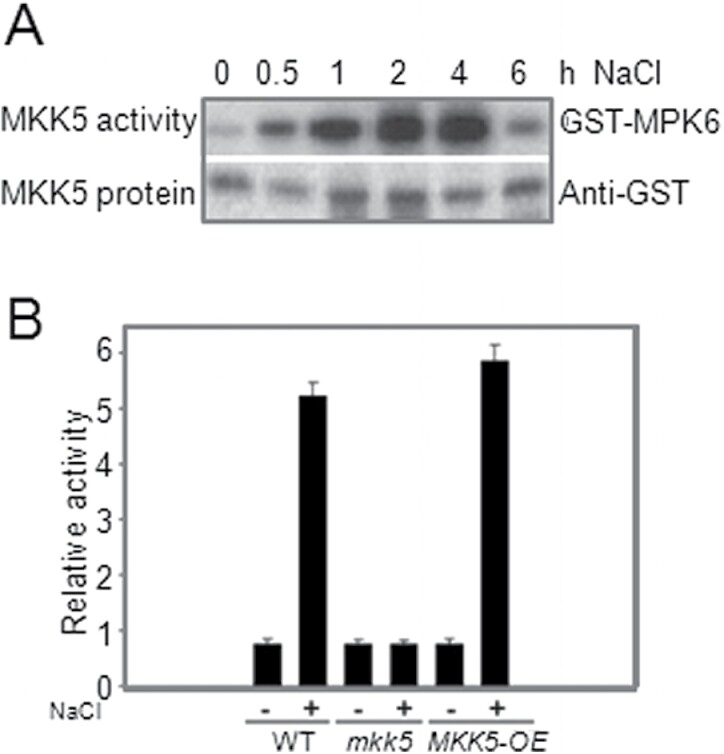
Activation of MKK5 by salt stress. (A) MKK5 activity was determined after transient expression in plant cells upon salt stress. GST epitope-tagged MKK5 was immunoprecipitated from *Arabidopsis* protoplasts following NaCl (150mM) treatments for 10min. MKK5 kinase activity was determined by *in vitro* kinase assays using kinase inactive GST-MPK6 as a substrate. (B) Relative *GUS* activity driven by the *MKK5* promoter in wild-type, *mkk5*, and MKK5-overexpressing plants induced by NaCl. The *MKK5* promoter was fused to *GUS* and tested for its response to NaCl in transient expression assays using protoplasts. All experiments were repeated at least three times with similar results.

Salt activation of the *FSD2* and *FSD3* promoters was not observed in *mkk5* protoplasts, so it was examined whether NaCl could activate MKK5 in *Arabidopsis* leaves. MKK5 activity was present from 0.5 to 6h after treatment and then dramatically decreased to basal levels (Fig. 4A). To confirm these results, *MKK5*-overexpressing plants (*MKK5*-OE) were constructed, and, consistent with the results using protoplasts, NaCl activation of the *MKK5* promoter was absent in *mkk5* but increased in *MKK5*-OE plants (Fig. 4B). These studies also demonstrated that the *Arabidopsis* protoplast transient expression system can offer a rapid and reliable tool for studying NaCl-induced FSD signalling based on early gene transcription.

### *MKK5*-overexpressing plants exhibit salt tolerance phenotypes

**Fig. 5.**
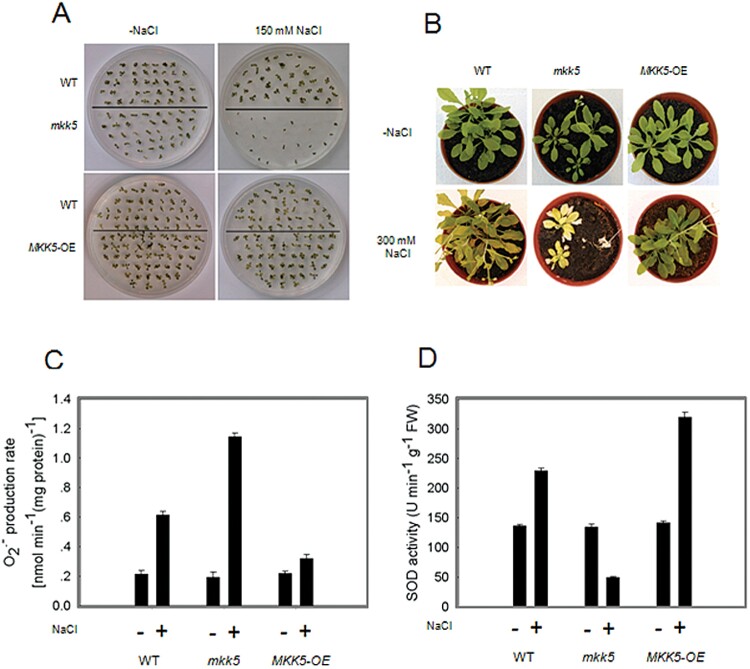
Phenotypic analysis of and overexpressing plants. (A) The salt-sensitive phenotype of *MKK5*-RNAi plants, *mkk5*, was investigated by germination assays of wild-type [Col-0 (WT)], *mkk5*, and *MKK5* overexpressing plants on agar plates, with 0mM or 150mM NaCl. Seeds were sterilized, stratified, and plated. Germination was visually determined after10 d. (B) Salt-sensitive phenotype of *mkk5* plants and salt tolerance of *MKK5* overexpressing lines. (C) The production of superoxide in seedlings response to salt stress (300mM NaCl) for 10 d. (D) SOD activities in seedlings of wild-type, *mkk5*, and *MKK5-OE* response to salt stress (300mM NaCl) for 10 d. All experiments were repeated at least three times with similar results.

The *MKK5*-OE plants showed no obvious phenotype under normal conditions, but large differences when they were stressed with salt treatments (Fig. 5B). To analyse salt tolerance of the *MKK5*-OE and *mkk5* plants, their germination efficiency on salt-containing media was evaluated. The *mkk5* plants germinated at a much lower rate than either wild-type or *MKK5*-OE, while the latter showed a slightly improved ability to germinate on salt-containing media (Fig. 5A). A quantification of the differences in germination of the different lines on salt-containing media confirmed the qualitative analysis: the germination rate of the *mkk5* plants was only 50% of that of wild-type. As shown in Fig. 5B, *MKK5*-OE plants exhibited increased salt tolerance compared with wild-type and the *mkk5* plants, so while wild-type and *MKK5*-overexpressing plants survived the salt stress conditions, the *mkk5* plants were sensitive to salt stress. Notably, the increased superoxide production was relatively much lower in *MKK5*-overexpressing plants than in the *mkk5* and wild-type plants, and SOD activity was also higher in *MKK5*-overexpressing plants (Fig. 5C, D). Similar results were obtained with 10 independent *MKK5*-OE lines in at least three independent assays. Taken together, these results confirm the importance of *MKK5* in conferring salt stress tolerance.

### MEKK1 interacts with MKK5

**Fig. 6.**
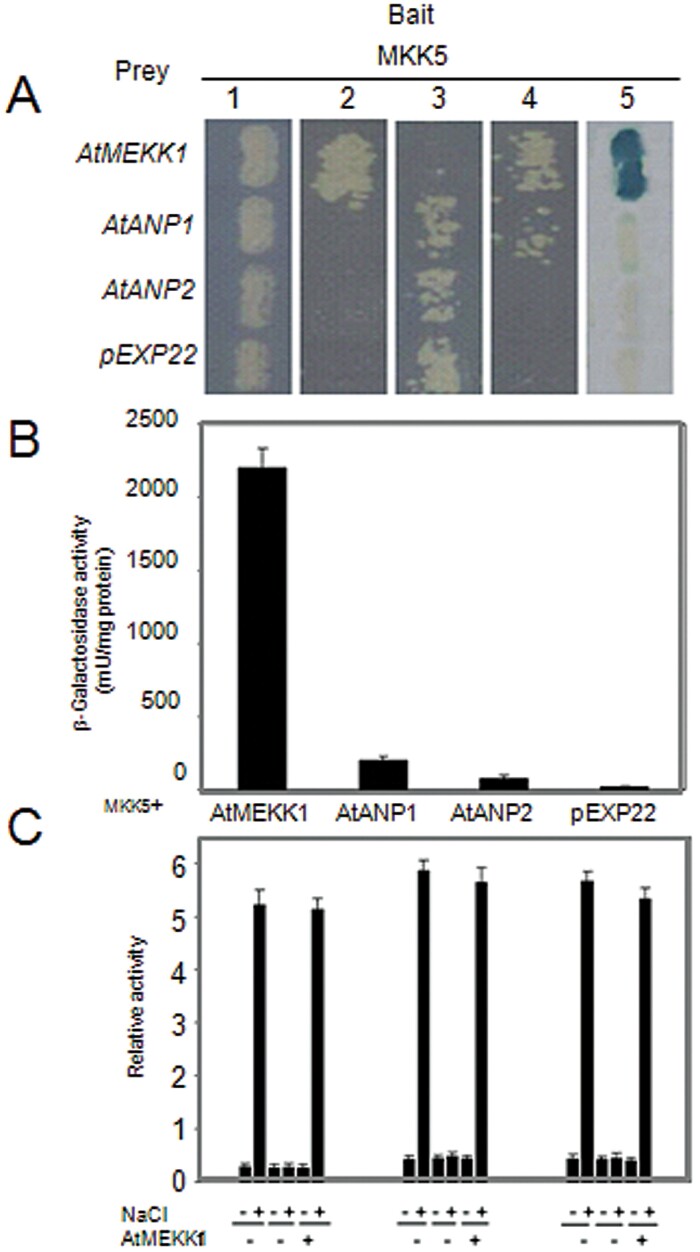
MKK5 specifically interacts with MEKK1. (A) MKK5 strongly interacted with MEKK1 in the yeast two-hybrid system. Yeast strains containing pEXP^TM^32-MKK5 as bait and pEXP^TM^22-MEKK1 as prey were grown on the following medium to screen for transformants: 1, SC medium lacking Leu and Trp for 48h; 2, SC medium lacking Leu, Trp, and Ura for an additional 48h to select the transformants; 3, SC medium lacking Leu and Trp and adding 0.2% of 5-fluoroorotic acid (5-FOA) for an additional 48h to select the cells containing interacting proteins; 4, SC medium lacking Leu, Trp, and His and adding 100mM 3-Amino-1,2,4-Triazole (3AT) for an additional 48h to confirm the interaction; 5, YPAD medium 48h, then an X-Gal assay was performed on the membrane to confirm the results. The pEXP^TM^22 empty prey vector was used as negative control. (B) Quantitative analysis of -galactosidase activity of the yeast strains in liquid culture showing the interaction between MKK5 and MEKK1. Values are means of data from at least three independent experiments. (C) Relative *GUS* activity driven by *MKK5*, *FSD2*, or *FSD3* promoters, respectively, showed the response to NaCl in transiently transfected protoplasts of wild-type (M) or *mekk1* (*m*) plants. The *MEKK1* genes were cloned and co-transformed into protoplasts of the *mekk1* mutant. All experiments were repeated at least three times with similar results.

The *MEKK1* gene has previously been shown to be upregulated by both cold and salt stresses (Mizoguchi *et al.*, 1997; Teige *et al.*, 2004) and it has also been shown that the MEKK1MKK4/5MPK3/6 module acts downstream of the flagellin receptor FLS2 and upstream of the WRKY22 and WRKY29 transcription factors (Asai *et al.*, 2002). Having established that MKK5 is involved in salt-induced FSD signalling, it was hypothesized that MKK5 might interact with MEKK1 under salt stress conditions. It was examined whether MKK5 and MEKK1 interact physically using the yeast two-hybrid system. Yeast strains containing pEXP-MKK5 were used as a bait, and MEKK1, ANP1, and ANP2 were chosen as candidates for upstream MKK5 action, since they have been shown to be activated by oxidative stress (Kovtun *et al.*, 2000; Krysan *et al.*, 2002). As can be seen in Fig. 6A, MEKK1 interacted strongly with MKK5 but not with ANP1 or ANP2. The coloured product produced in the assay appeared and this was confirmed by a quantitative -galactosidase assay (Fig. 6B). Finally, it was tested whether MKK5, the transcript levels of FSD2, and FSD3 were affected in wild-type and the *mekk1* mutant, by fusing their promoters to *GUS* and testing their response to NaCl in transiently transfected protoplasts of wild-type and *mekk1* mutant. Consistent with the results from the yeast two-hybrid assay, the activation of *MKK5*, *FSD2*, and *FSD3* promoters were all arrested in the *mekk1* mutant (Fig. 6C), suggesting that MEKK1 acts upstream of MKK5 and is involved in NaCl-induced FSD signalling.

## Discussion

Salt stress can modulate the activity of the antioxidant system (Khan *et al.*, 2012), and several studies have demonstrated that antioxidant enzyme activities and levels of antioxidant compounds are increased in certain plants in response to salt stress (Meneguzzo *et al.*, 1999; Shalata *et al.*, 2001). As the first line of defence against ROS in plant cells, SODs react with the superoxide radical to produce H_2_O_2_ (Scandalios, 1997). SODs have been extensively studied for many years and growing evidence suggests that they respond to many abiotic stresses and that SOD overexpressing plants have an enhanced tolerance of abiotic stresses (Miller *et al.*, 2008, van Breusegem *et al.*, 2008; Li *et al.*, 2013; Xing *et al.*, 2013). This study was focused on FeSODs and analysing their response to salt stress. Of the three *FeSODs* from *Arabidopsis*, *FSD2* and *FSD3*, but not *FSD1*, were strongly regulated by salt stress. Possibly as a consequence of circadian regulation, the mRNA abundance of *FSD1* was not very stable but was normally higher than that of *FSD2* or *FSD3* (Fig. 1A). Different FeSODs respond to stresses in various ways, suggesting complex mechanisms for regulating the expression of the SOD gene family, involving substantial signalling pathways crosstalk.

The specific ROS sensors that process and translate associated stresses have yet to be identified (Andreasson and Ellis, 2010). However, the crosstalk among ROS signalling, ROS scavenging enzymes, and MAPK cascades is complex and elaborate. There is evidence from several systems for the interaction of MAPKs with ROS (Samuel and Ellis, 2002; Pitzschke and Hirt, 2006; Xing *et al.*, 2008, 2013). The MAPK signalling pathway, as one of the major signalling cascades, plays a crucial role in diverse cellular functions, and particularly the redox-regulated processes involved in cellular metabolism (Davletova *et al.*, 2004; Joo *et al.*, 2005; Pitzschke and Hirt, 2006; Xing *et al.*, 2008, 2013). In some studies, H_2_O_2_ is required for the activation of ZmMPK5 in maize leaves (Ding *et al.*, 2009; Lin *et al.*, 2009). ZmCPK11 induces the activation of ZmMPK5 in ABA signalling by increasing the production of H_2_O_2_ (Ding *et al.*, 2013). Cai *et al.* (2014) found overexpression of *ZmMKK1* alleviated ROS accumulation by maintaining high activities of ROS scavenging enzymes such as SOD, POD, CAT, and APX. Similarly, *ZmMPK17* and *ZmMKK4* transgenic tobacco plants have enhanced osmotic stress tolerance through high ROS scavenging ability (Kong *et al.*, 2011;).

Although both MAPK signalling cascades and antioxidant systems are involved in biotic and abiotic stress, it is not clear how or if they interact. In a systematic screen of the 10 members of the *Arabidopsis* MAPKK family, it was found that transcription of the NaCl-activated *FSD2* and *FSD3* was significantly blocked in *MKK5*-RNAi plants*mkk5*but not in *mkk4*the *MKK4*-RNAi plants, which belongs to the same subfamily as MKK5. In the flagellin-induced pathway of the MEKK1MKK4/MKK5MPK3/MPK6 module, *MKK4* and *MKK5* are involved in the same process of regulating the downstream WRKY22 and WRKY29 transcription factors (Asai *et al.*, 2002). MKK4 and MKK5 are paralogous MKKs acting upstream of the MPK3/MPK6, and play a key role in mediating many different stress signals and in plant development (Andreasson and Ellis, 2010). It is very interesting that MKK5 and MKK4 show different responses to salt stress, while MKK4 was not involved in the regulation of FSD (Fig. 2A). The present study reveals that MKK4 and MKK5 may operate in different pathways and that only MKK5 is involved in NaCl-induced salt stress. The regulation of MKK5 on FSD2/3 seems to be specific. The function of MKK4 in salt stress, in contrast to MKK5, was investigated, and the salt tolerance in MKK4-RNAi plants tested. The MKK4-RNAi line did not show similar phenotypes as MKK5-RNAi plants under salt stress (data not shown). Miles *et al.* (2009) also showed that partial suppression of MKK5 expression in *Arabidopsis* is sufficient to induce ozone hypersensitivity, which would indicate that MKK4 and MKK5 are not fully redundant. However, it is still possible that the roles of MKK4 and MKK5 within the salt stress signalling network are redundant because there is no experimental evidence of a double mutant at present.

The NaCl-induced MAPK cascade leading to the activation of *Arabidopsis MPK3*, *MPK4*, and *MPK6* is reminiscent of the activation of *MPK4* and *MPK6* by *SIMK*, under salt stress (Kiegerl *et al.*, 2000). Teige *et al.* (2004) identified a salt-induced *Arabidopsis* MAPKK, *MKK2*, which is capable of activating *MPK4* and *MPK6.* However, as shown in Fig 3A, these data further revealed an unexpected activation of *MPK3*. It was also shown that although *MPK3*, *MPK4*, and *MPK6* are involved in salt stress responses, only *MPK3* and *MPK6* are regulated by *MKK5* (Fig. 3C). In contrast to the activation of *MPK4* and *MPK6* by *MKK2* following salt stress, the regulation of *MPK3* and *MPK6* by *MKK5* is more complex, involving different MAPK signalling pathways. The MKK5MPK3/MPK6 pathway is similar to the flagellin-induced MAPK signalling pathway, MKK4/MKK5MPK3/MPK6 (Asai *et al.*, 2002). The existence of a crosstalk between the different stress signals is suggested, and that MKK5, as an important component of MAPKs signalling cascades, plays a key role in plant responses to biotic and abiotic stresses. The MAPK family involves comprehensive protein interactions and different MAPKs may function to different stresses (Andreasson and Ellis, 2010). Together these data demonstrate that MKK5 is a key signal transducer in MAPKs signalling cascades involved in regulating different processes in response to multiple divergent stresses.

## Supplementary data

Supplementary material is available at *JXB* online.

**Figure S1.** Original images of RNA gel blots used for preparation of Figure 1A. (A) FSD1 expression. (B) FSD2 expression. (C) FSD3 expression. (D) Actin gene ACT2 was used as loading control.

**Figure S2.** Original images of RNA gel blots used for preparation of Figure 2A. (A) FSD2 expression. (B) FSD3 expression. (C) Actin gene ACT2 was used as loading control.

**Figure S3.** Original images of MAPK activity analysis used for preparation of Figure 3A. (A) MAPK activity of control, MPK2 and MPK3. (B) MAPK activity of MPK4, MPK6, MPK7 and MPK9.

**Figure S4.** Original images of MAPK activity analysis used for preparation of Figure 3B. (A) MAPK activity of MPK3, MPK4 and MPK6 in WT. (B) MAPK activity of MPK3, MPK4 and MPK6 in *mkk5* mutant. (C) MAPK activity of MPK3, MPK4 and MPK6 in *MKK5*-OE plants.

**Figure S5.** Original images of MKK5 activity analysis used for preparation of Figure 4A. (A) MKK5 activity. (B) Western Blot analysis of GST epitope-tagged MKK5.

erv305_suppl_Supplementary_Figures

## Abbreviations:

CaMV: cauliflower mosaic virus
FeSOD: iron SOD
FSD: iron superoxide dismutase
GUS: -glucuronidase
MAPK: mitogen-activated protein kinase
MAPKK: MAPK kinase
4-MU: 4-methyl-umbelliferone or 7-hydroxy-4-methyl-cumarin
MUG: 4-methyl-umbelliferyl--D-glucuronide
ROS: reactive oxygen species
RT-PCR: reverse transcription-PCR
SIMK: stress-induced MAPKK
SIMKK: stress-induced MAPKK
SOD: superoxide dismutase
XTT: 3-[1-(phenylamino-carbonyl)-3,4-tetrazolium]-bis(4-methoxy-6-nitro) benzenesulphonic acid hydrate.
