# Corrigendum to: Mitogen-activated protein kinase kinase 5 (MKK5)-mediated signaling cascade regulates expression of iron superoxide dismutase gene in *Arabidopsis* under salinity stress

**DOI:** 10.1093/jxb/erab133

**Published:** 2021-05-06

**Authors:** Yu Xing, Wei-Hua Chen, Wensuo Jia, Jianhua Zhang

**Affiliations:** 1 Beijing Key Laboratory for Agricultural Application and New Technique and College of Plant Science and Technology, Beijing University of Agriculture, Beijing, China; 2 Institute of Agro-Products Processing Science & Technology CAAS, Beijing, China; 3 College of Agronomy and Biotechnology, China Agricultural University, Beijing, China; 4 School of Life Sciences, State Key Laboratory of Agrobiotechnology, and Shenzhen Research Institute, The Chinese University of Hong Kong, Hong Kong, China

*Journal of Experimental Botany*, Vol. 66, No. 19 pp. 5971–5981, 2015, doi:10.1093/jxb/erv305

In the above article, cropped and contrast-adjusted images were used to prepare some of the figures. The authors have now added the original images as Supplementary Figures S1–S5.

