# Correction

**DOI:** 10.1093/jxb/erac332

**Published:** 2022-09-14

This is a correction to: Yu Xing, Wei-Hua Chen, Wensuo Jia, Jianhua Zhang, Corrigendum to: Mitogen-activated protein kinase kinase 5 (MKK5)-mediated signaling cascade regulates expression of iron superoxide dismutase gene in *Arabidopsis* under salinity stress, *Journal of Experimental Botany*, Volume 72, Issue 13, 22 June 2021, Page 5094, https://doi.org/10.1093/jxb/erab133

Yu Xing, Wei-hua Chen, Wensuo Jia, Jianhua Zhang, Mitogen-activated protein kinase kinase 5 (MKK5)-mediated signalling cascade regulates expression of iron superoxide dismutase gene in *Arabidopsis* under salinity stress, *Journal of Experimental Botany*, Volume 66, Issue 19, September 2015, Pages 5971–5981, https://doi.org/10.1093/jxb/erv305

During the preparation of the corrigendum, the authors mistakenly included the wrong original image (Figure S5B) for MKK5 protein used in Figure 4A. The correct original is now available in the supplementary file (Figure S5B) in the online version of the article (https://doi.org/10.1093/jxb/erv305).

