# Supplementary material for: Mitogen-activated protein kinase kinase 5 (MKK5)-mediated signalling cascade regulates expression of iron superoxide dismutase gene in Arabidopsis under salinity stress
Source: J Exp Bot. 2015 Jul 1;66(19):5971–81. doi: 10.1093/jxb/erv305 (PMC4566985; doi:10.1093/jxb/erv305)
Supplement: erv305_suppl_Supplementary_Figures [file erv305_suppl_supplementary_figures.pdf]

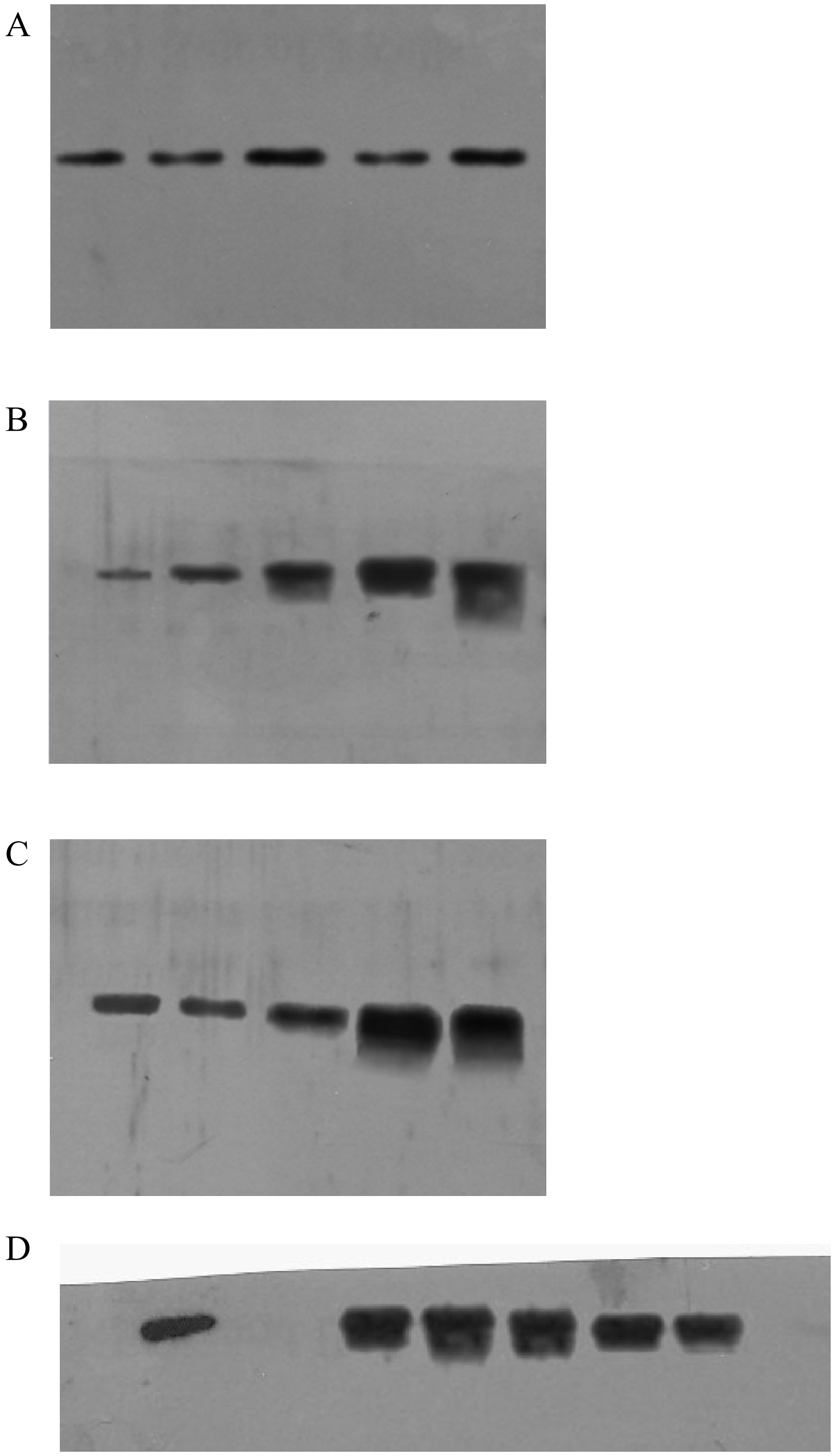


**Figure S1.** Original images of RNA gel blots used for preparation of Figure 1A. (A) FSD1 expression. (B) FSD2 expression. (C) FSD3 expression. (D) Actin gene ACT2 was used as loading control.

A

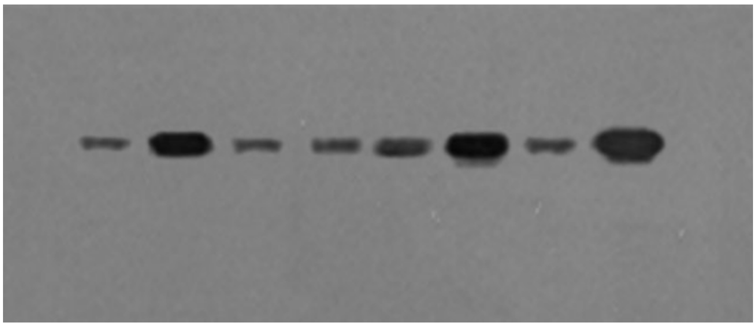

B

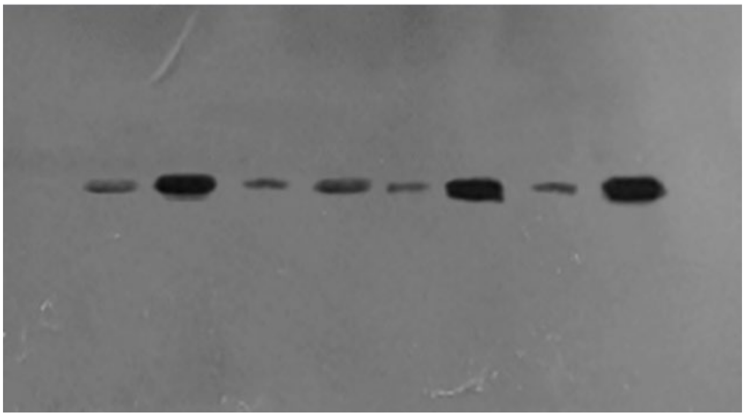

C

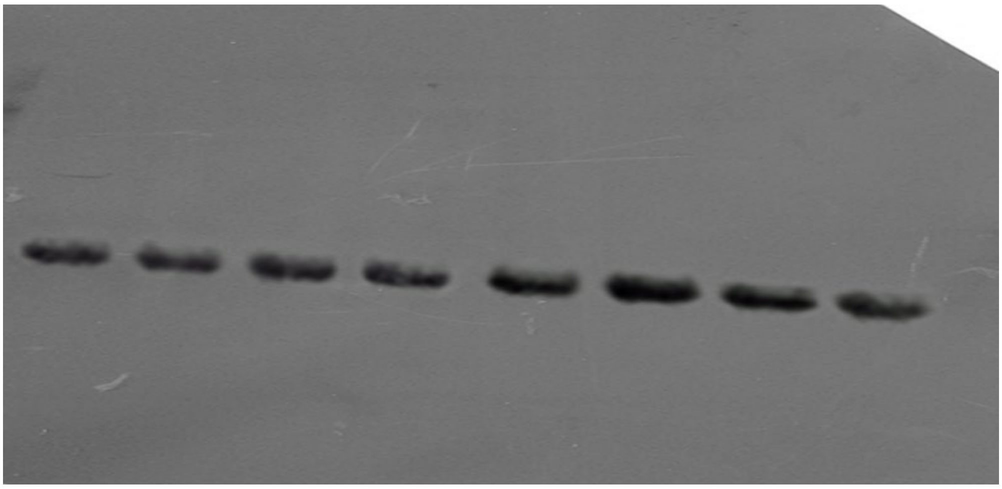

**Figure S2.** Original images of RNA gel blots used for preparation of Figure 2A. (A) FSD2 expression. (B) FSD3 expression. (C) Actin gene ACT2 was used as loading control.

A

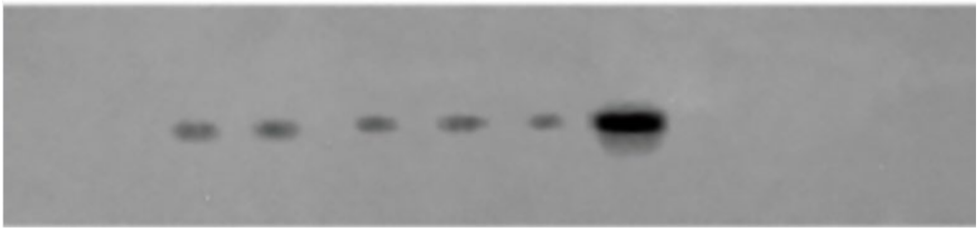

B

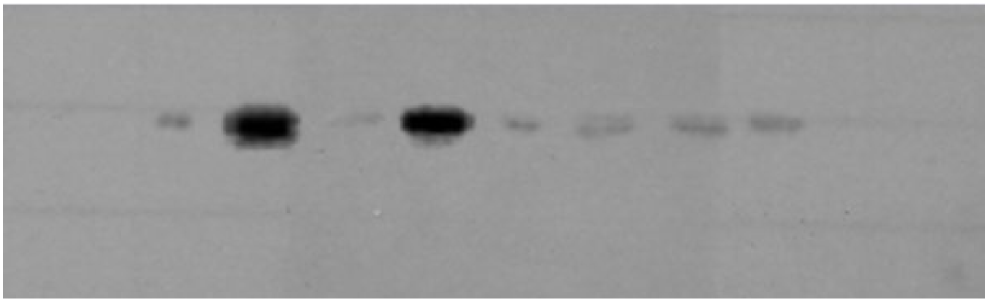

**Figure S3.** Original images of MAPK activity analysis used for preparation of Figure 3A. (A) MAPK activity of control, MPK2 and MPK3. (B) MAPK activity of MPK4, MPK6, MPK7 and MPK9.

A

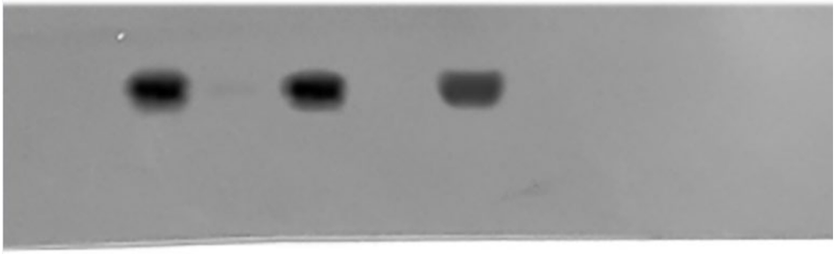

B

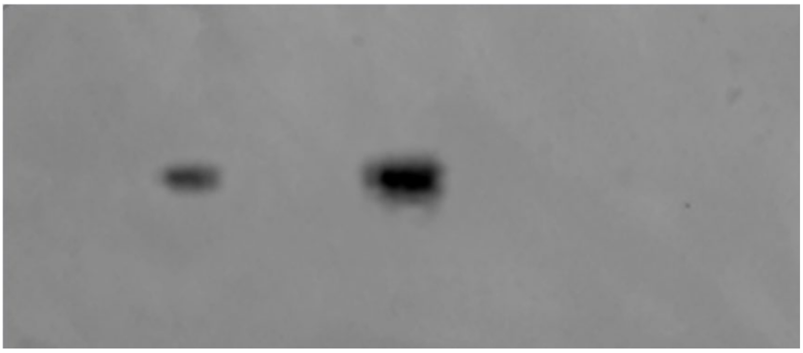

C

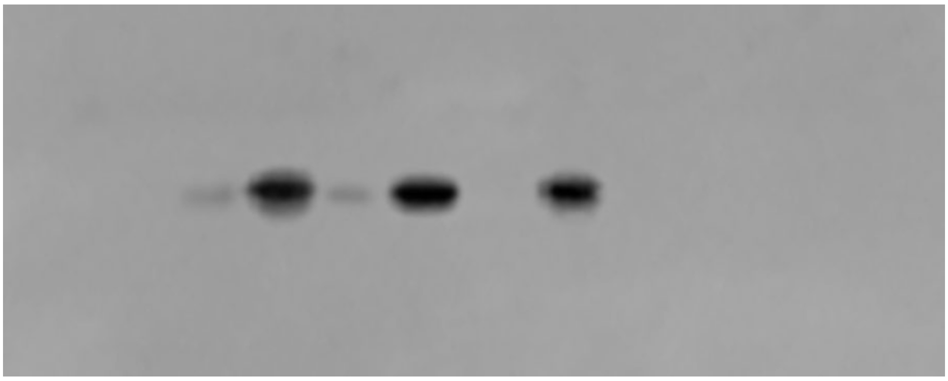

**Figure S4.** Original images of MAPK activity analysis used for preparation of Figure 3B. (A) MAPK activity of MPK3, MPK4 and MPK6 in WT. (B) MAPK activity of MPK3, MPK4 and MPK6 in *mkk5* mutant. (C) MAPK activity of MPK3, MPK4 and MPK6 in *MKK5*-OE plants.

A

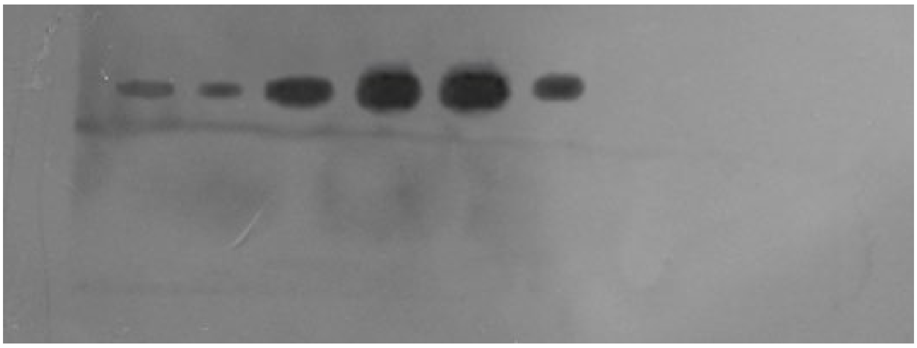

B

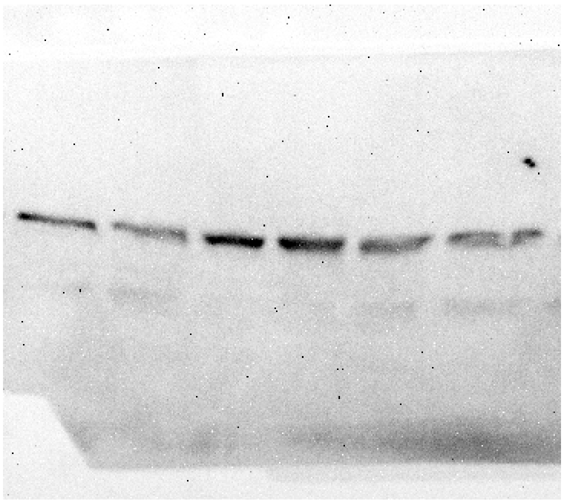

**Figure S5.** Original images of MKK5 activity analysis used for preparation of Figure 4A. (A) MKK5 activity. (B) Western Blot analysis of GST epitope-tagged MKK5.
